# New Gene Targets for Diagnosis and Therapy of Diabetic Retinopathy

**DOI:** 10.5152/eurasianjmed.2025.24559

**Published:** 2025-04-19

**Authors:** Emine Çinici, Mehmet Enes Arslan, Özge Çağlar Yıldırım, Nilay Dilekmen, Bahadır Utlu, Özkan Çinici, Zehra Sağlam, Hasan Türkez

**Affiliations:** 1 Department of Ophthalmology, Atatürk University Faculty of Medicine, Erzurum, Türkiye; 2 Department of Molecular Biology and Genetics, Erzurum Technical University Faculty of Science, Erzurum, Türkiye; 3 Department of Ophthalmology, Palandöken State Hospital, Erzurum, Türkiye; 4 Department of Ophthalmology, Regional Training and Research Hospital, Health Sciences University Faculty of Medicine, Erzurum, Türkiye; 5 Department of Internal Medicine, Regional Training and Research Hospital, Erzurum, Türkiye; 6 Department of Medical Biology, Atatürk University Faculty of Medicine, Erzurum, Türkiye

**Keywords:** Candidate genes, diabetic retinopathy, gene–disease relationship, gene expression analysis, neurodegeneration

## Abstract

**Objective:**

Diabetic retinopathy (DR), considered one of the most common microvascular complications associated with diabetes mellitus (DM), involves both neuronal and vascular dysfunctions in the retina. Neuronal damage and vision loss occur progressively in patients with DR. A number of genetic targets have been identified for DR and gene-related treatments as well as early diagnostic techniques have been developed. Despite some medical advances, DR remains a devastating complication of diabetes. This study aimed to identify new gene targets that can be used for the prognosis and treatment of DR..

**Materials and Methods:**

Eight candidate genes were analyzed using Synergy Brands Green (SYBR-green)-based real-time polymerase chain reaction in peripheral blood mononuclear cells (PBMCs) from 45 individuals: DR patients (n = 15), DM patients without DR (n = 15), and healthy controls (n = 15). STRING v11 was used for protein–protein interaction analysis. Gene expression differences were evaluated using ANOVA, with significance set at *P* < .05.

**Results:**

HIF1A and VEGFA were significantly upregulated in both DR and DM groups compared to controls (HIF1A: fold change 5.28; VEGFA: fold change 5.20 for DR group). SERPING1 was specifically upregulated in DR patients (fold change 3.42). CX3CR1 and BDNF were downregulated in both DR and DM groups (CX3CR1: fold change 8.32; BDNF: fold change 3.21), while IGFBP3 was significantly downregulated only in DR patients (fold change 6.5). STRING analysis revealed strong interactions between SERPING1 and complement pathway components, while IGFBP3 was linked to insulin-like growth factor signaling.

**Conclusion:**

In light of these findings, we observed that SERPING1 and IGFBP3 genes might be proposed as targets for early diagnosis and treatment for DR.

**Main Points**

- In this article, new target genes were identified for the diagnosis and treatment of DR through gene expression analysis.
- HIF1A and IGFBP3 genes were identified as potential markers to confirm retinopathy progression in diabetic patients.
- HIF1A upregulation and IGFBP3 downregulation were associated with the transition from DM to DR pathogenesis.

## Background

Diabetes mellitus (DM) is one of the most critical health problems worldwide. While insulin production is not sufficient in Type I of the disease, cellular insulin resistance occurs in Type II. Both types of disease are associated with hyperglycemia, oxidative stress, inflammation, macrovascular, and microvascular (e.g., retinopathy and nephropathy) diseases. The number of DM patients is increasing rapidly every year. The World Health Organization (WHO) reports that by 2025, the number of people affected by the disease and impaired glucose tolerance will reach to 418 million.^1^

Diabetic retinopathy (DR) is considered one of the most important causes of blindness among adults aged 20-74 years in developed countries. It is estimated that the rate of DM patients worldwide is about 1.5-2%. Approximately 25% of these patients have DR at any stage. Diabetic retinopathy is a microangiopathy affecting precapillary arterioles, capillaries, and venules in the retina. Chronic hyperglycemia can cause an increase in intracellular sorbitol, glycosylation of intracellular lipids and proteins, and an increase in glycation end products, oxidative stress, inflammation by activating the polyol pathway, causing damage to the endothelium and pericyte cells, and the development of retinopathy.^2^

Several genetic studies and genome-wide association studies (GWAS) have proposed associations between DR and genetic variants of the aldose reductase (*ALR2*), thereceptor for advanced glycation end product gene (*RAGE*), and vascular endothelial growth factor (*VEGF*) genes.^3^ A previous study by the Group of Diabetes Control and Complications Trial Research indicated that genetic factors were effective in the initiation of retinopathy in diabetic patients.^3^ A recent study reported that neurodegeneration-related genes, including *MAPT*,* ROCK2*, and *UHRF1*, might be responsible for early pathological alterations in patients with DR.^4^ Besides, genes including *AIF1*,* ALDH3A1*,* COL1A1*,* FGF23*,* ITGA7*,* MAPK13*,* PIK3CB*,and *THBS1* serve crucial functions in angiogenesis-related pathways, and they have been suggested as candidate genes in the early development of DR.^5^ In sum, DR-related genes were involved in critical processes such as insulin signaling, angiogenesis, inflammation, neurogenesis, regulation of endothelial cell/leukocyte interaction, and neurodegeneration. However, these results were obtained from genetic-based studies and have not been validated in multiple human populations. Hence, biomarkers for genetic risk indication toward DR were suggested to determine disparately in other ethnic groups.^6^

Several reports have documented the presence of remarkable microvascular abnormalities due to damage to retinal neurons in patients with DR at early stages; in this respect, DR is considered as a neurodegenerative disease of the retina.^7^ At this point, elucidation of the molecular genetic mechanisms underlying neurodegeneration in DR and identifying genes involved in neurodegeneration during the progression of DR is critical for developing novel prognostic and therapeutic options.^8^ Therefore, in the present study, we aimed to reveal the expression profiling of 8 candidate genes in patients with DR and DM compared to healthy people. Eight candidate genes, including brain-derived neurotrophic factor (*BDNF*), chemokine fractalkine (CX3CL1)/CX3C chemokine receptor-1 (*CX3CR1*), hypoxia-inducible factor-1α (*HIF1A*), insulin-like growth factor-binding protein-3 (*IGFBP3*), serine (or cysteine) proteinase inhibitor, clade G (C1 inhibitor) (*SERPING1*),signal transducer and activator of transcription-3(*STAT3*),tissue inhibitor of metalloproteinase-3(*TIMP3*), and vascular endothelial growth factor-a (*VEGFA*), were found to have critical functions in neurodegenerative processes.^9^ Thirty patients with DR and DM and 15 healthy subjects were included in this study, and blood samples were collected to conduct gene expression profiling analysis. The differentially expressed genes were associated through STRING v11: protein–protein association networks online tool to identify gene-to-gene relationships.

## Materials and Methods

### Experimental Groups and Patients

A total of 45 people participated in this study. To assess early indicators of the disease, non-proliferative DR patients were studied. The first group (n = 15) consisted of DR patients with a mean age of 51.3 registered in the ophthalmology clinic and they did not receive anti-VEGF therapy. The second group consists of DM patients with a mean age of 49.3. These patients (n = 15) were registered at the internal medicine clinic for routine control. Moreover, the control group consisted of 15 subjects with a mean age of 49.7 without any disease and did not smoke. The participants in the study did not have any known metabolic conditions, such as obesity or hypertension. The baseline characteristics of the participants are presented in Table 1. Socio-demographic and clinical details of the patients and healthy individuals were recorded by submitting the standard questionnaire at the recruitment time. This study was conducted in accordance with the Declaration of Helsinki with the approval of the Ethics Committee of Atatürk University (Approval No: 51; Date: 22.04.2019). Written informed consents were obtained from all subjects.

### Peripheral Blood Mononuclear Cells Isolations

Ten milliliter blood sample was taken from the participants for peripheral blood mononuclear cells (PBMCs) isolation. Isolations were performed using the Ficoll density method. Whole blood was mixed with Fetal Bovine Serum (PBS) in a ratio of 1:1. Then, 15 mL of Ficoll was taken into a new tube. Blood diluted with PBS was gently dropped onto Ficoll. The tube was centrifuged at 18-24°C, 400 g for 30 minutes. The PBMCs layer was taken into a new tube, and then 5 mL of PBS was added. The tube was re-centrifuged at 18-24°C, 100 g for 10 minutes. Then supernatant was then removed, and the pellet was washed with PBS for further steps.^10^

### Ribonucleic Acid Isolation and Complementary Deoxyribonucleic Acid Synthesis

Total ribonucleic acid (RNA) isolation was performed from PBMCs. PureLink™ RNA Mini Kit (Invitrogen, USA) was used for this purpose. At the end of the method, following the kit procedure, a UV-visible spectrophotometer (NanoDrop, USA) and a bioanalyzer (Agilent Technologies, USA) were used to evaluate the RNA purity and quality. The isolated RNAs were stored at −20°C until the next study. Complementary deoxyribonucleic acid was synthesized from total RNA following the manufacturer’s (QuantiTect Reverse Transcription Kit, Qiagen) recommendation.

### Real-Time Polymerase Chain Reaction

In the study, the expression profile of a total of 8 genes including *BDNF, CX3CR1, HIF1A, IGFBP3, STAT3, VEGFA, TIMP3, *and *SERPING1* was investigated with the real-time polymerase chain reaction (PCR) technique. For this purpose, the used primers were presented in Table 2. Real-time PCR was performed by the SYBR-green method.^11^ The kit procedure (QuantiTect SYBR Green PCR Kit) was followed by the analysis performed using 100 ng RNA samples. To validate a single PCR product presence, a melting curve analysis was performed. The cut-off value for the cycle threshold (CT) analysis was selected as 35 cycles and the threshold was set over the background signal and near the middle of the logarithmic phase of the amplification plot. The Livak (2^−ΔΔCt^) method was used to calculate relative gene expression, with CT values normalized to the GAPDH reference gene.^12^

### Statistical Analysis

In this study, GraphPad Prism^®^ version 7.0 software was used to obtain statistical data analysis for the performed experiments. One-way ANOVA and Tukey analysis were used for comparing each data set, and statistically significant level was chosen as 95% (*P* < .05). The gene–disease relationship was investigated using STRING v11 multiple protein comparison analysis for both DR and DM patients.^13^ G*Power (Version 3.1.9.7) was used to calculate the power of the study above 0.9 with a significance level of 0.5%.

## Results

**Figure 1.**
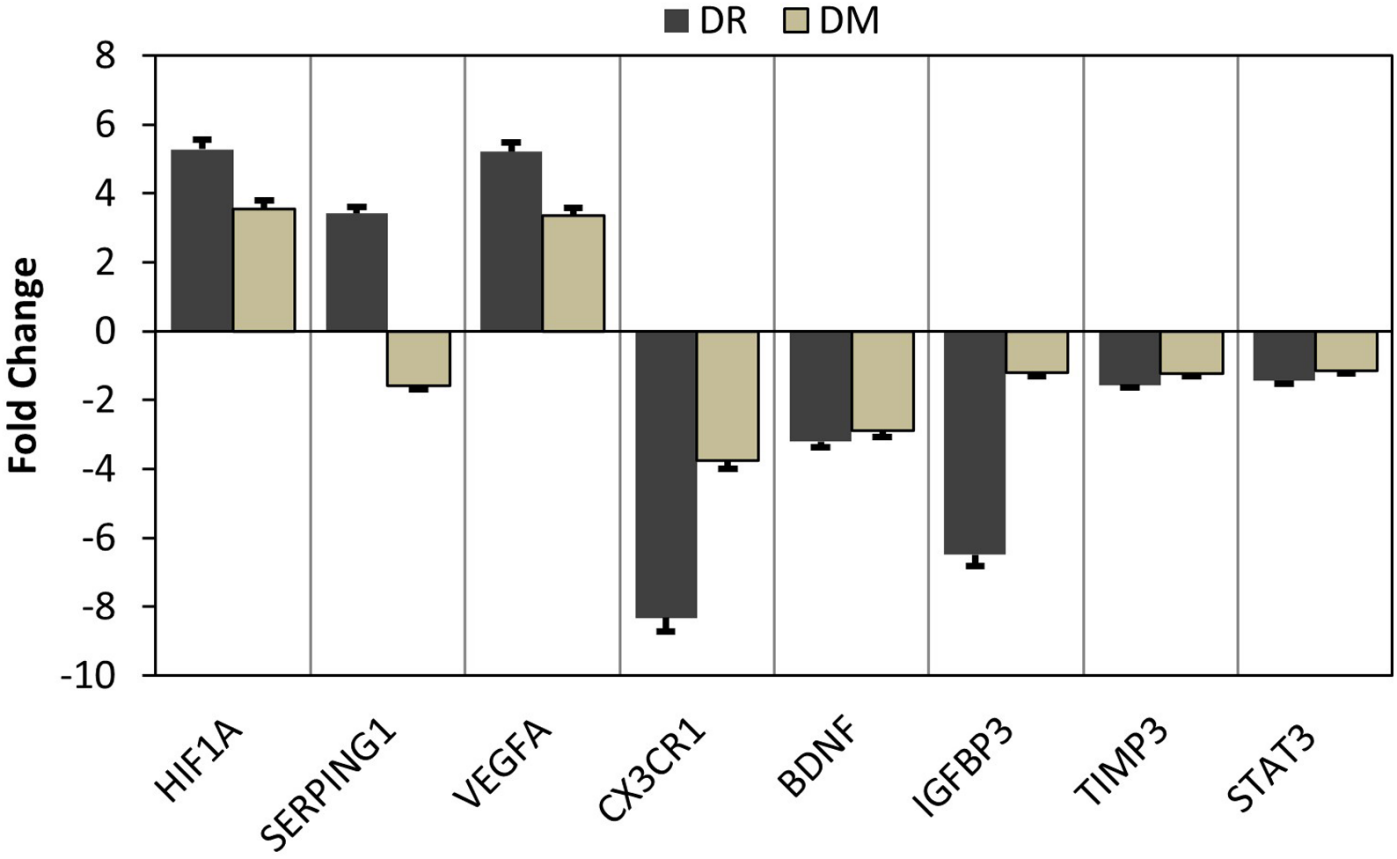
Gene expression analysis of diabetic retinopathy and diabetes mellitus groups compared to control subjects. Symbol (*) represents a statistically significant increase or decrease in gene expression compared to control subjects (fold change ≥ 2).

Three study groups with DR and DM patients and healthy people were recruited in this study. Peripheral blood mononuclear cell were isolated from the participants to isolate total RNA for gene expression analysis. SYBR-green-based real-time PCR technique was used to investigate the differentially expressed genes for each case. According to the gene expression analyses, we found that *HIF1A* and *VEGFA* genes were upregulated in both DR and DM patients compared to control subjects. We also found that *SERPING1* gene expression was upregulated only in DR patients. On the other hand, we found that *CX3CR1* and *BDNF* genes were downregulated for both DR and DM patients compared to control subjects. *IGFBP3* gene expression was found to be downregulated only in patients with DR (Figure 1). Sample size analysis was applied using power investigation (G*Power software) to confirm sample numbers were adequate for the gene expression analysis (Table 3).

**Figure 2.**
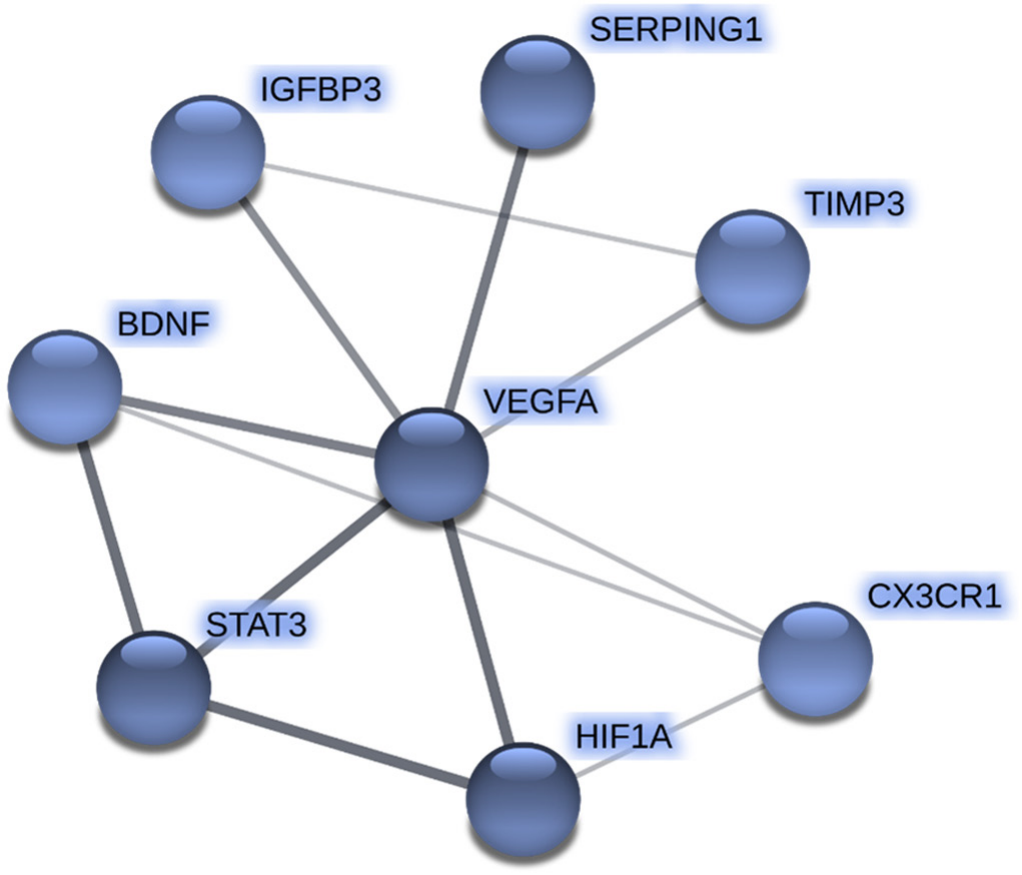
Protein–protein interaction network analysis (STRING v11) of differentially expressed genes in patients with diabetic retinopathy and diabetes mellitus.

Moreover, protein–protein interaction network analysis (STRING v11) revealed that all of the 8 candidate genes were connected to the *VEGFA* gene, which is known to be closely related to both DR and DM patients. In addition, there was no direct connection between the differentially expressed genes, which were *SERPING1* and *IGFBP3* genes (Figure 2).

## Discussion

There has been a significant effort to develop gene-based diagnostic tests to predict drug response, choose the best treatment option, and diagnose diseases at earlier stages. The eye as an organ is a good candidate for targeted therapy because of its strong compartmentalization, privileged status in immunity, tissue transparency, and accessibility. Additionally, it is an ideal model for randomized control trials and translational studies to obtain a better outcome analysis.^14^ Moreover, finding signatures in gene expression patterns may provide a powerful drug discovery and disease diagnosis mechanism. For instance, gene expression analysis is being widely utilized in oncology concerning classification and diagnosis for patients with different cancer types such as kidney, breast, and prostate.^15^ Besides, gene-based therapies have been developed to regulate specific gene expression for disease condition therapy by delivering exogenous genetic material to patients. This concept provides stable and targeted gene expression regulation by overexpression or silencing of gene expression with a final therapeutic result and was originally designed for gene-specific applications.^16^

Our gene expression analysis revealed that the expression of *HIF1A* and *VEGFA* was upregulated in both DR and DM patients. Impairment in oxygen delivery to photoreceptors and the retinal pigment epithelium, which results from hypoxia, can affect cell function and metabolism and, ultimately, disturb cell survival. Supporting our data, previous studies exerted that overexpression of *HIF1A* might lead to slowly progressing retinal degeneration and neuronal cell death.^17^ In parallel with our findings, a remarkable increase in the level of *HIF1A *expression was determined in hypoxic human retinal glial (MIO-M1) cells.^18^ Furthermore, in accordance with our findings, it was known that *VEGFA* gene expression was intimately related to the pathogenesis of DR. Higher expression of the *VEGFA* gene was found to result in DR and recently, anti-VEGF treatment for DR cases was recommended instead of laser photocoagulation and intravitreal steroid treatments.^19^ Additionally, *VEGF* gene polymorphism was found to be closely related to the severity of DR through the analysis of single-nucleotide polymorphisms (SNPs).^20^ Our study also correlates with the literature in that *VEGF* gene expression was found to have a higher expression level in the DR group than in the DM patient group.

On the other hand, we found that the *SERPING1* gene was upregulated only in patients with DR. In agreement with the available literature, the *SERPING1* gene that encodes the C1INH protein, an inhibitor of lectin pathways of complement activation, is closely related to neuronal degeneration in the human eye.^21^ The increasing retinal expression of* SERPING1* led to the development of diabetic complications related to neurodegenerative, neurovascular, and inflammatory processes. Moreover, the SNP variant of the *SERPING1 *gene was found to be associated with macular degeneration-related blindness.^22^ However, the gene expression level of *SERPING1 *was not found to reach 2-fold changes. Thus, it would be further analyzed to relate DR and *SERPING1 *gene relationships. SERPING1, also known as C1 inhibitor, is a critical regulator of the classical complement pathway, modulating inflammatory responses. Given that inflammation and immune responses are pivotal in the pathogenesis of DR, the role of SERPING1 warrants thorough investigation. Recent studies have highlighted the involvement of the complement system in DR. For instance, Shim et al identified SERPING1 as a core gene associated with Type 2 DM, suggesting its potential involvement in DR development.^23^ Furthermore, researchers emphasized the integral association between the complement system and DR, proposing that complement activation contributes to retinal inflammation and vascular dysfunction.^24^ These findings underscore the importance of exploring SERPING1's function within the complement cascade to better understand its impact on DR progression. Future research focusing on SERPING1 and complement modulation may offer novel therapeutic strategies for DR management.

Our STRING v11 analysis revealed strong interactions between VEGFA and the other investigated genes (*HIF1A*, *CX3CR1*, *IGFBP3*, *STAT3*, *BDNF*, *TIMP3*, and *SERPING1*), highlighting the central role of VEGFA in the pathogenesis of DR and DM. The interaction between VEGFA and HIF1A supports the notion that VEGFA expression is upregulated under hypoxic conditions, thereby promoting retinal neovascularization.^17^ Additionally, the downregulation of CX3CR1 has been associated with microglial activation and inflammatory responses, potentially accelerating neurodegenerative processes.^25^ The observed link between IGFBP3 and VEGFA suggests that dysregulation of the IGF signaling pathway may contribute to retinal cell death, as previous studies have demonstrated that IGFBP3 deficiency leads to retinal vessel loss and neuronal degeneration.^5^ The interaction between STAT3 and BDNF may be linked to neuroprotective mechanisms, as reduced BDNF expression has been associated with increased oxidative stress in DR models.^26^ Furthermore, TIMP3's association with VEGFA suggests a role in regulating angiogenic processes, while the interaction between SERPING1 and VEGFA implies that complement system activation may contribute to retinal inflammation^21^,^22^ This network of interactions provides valuable insights into the multifactorial pathogenesis of DR and may facilitate the identification of novel therapeutic targets.

Our analysis showed that *CX3CR1* and *BDNF* gene expressions were decreased significantly in both DR and DM patients. However, *IGFBP3* gene expression was found to be reduced in DR patients. Indeed, chemokines and cytokines are essential factors for retinal degeneration and neuronal cell death because macrophage and microglia cell recruitment occur via the regulation of these factors. In line with the present results, *CX3CR1* gene knockout mice were shown to increase the accumulation of macrophages/microglia in the subretinal region, which is related to neuronal degeneration.^27^ Previous studies showed that retinal *BDNF* levels were reduced in diabetic animal models, and this was claimed to result from increased oxidative stress. Furthermore, it was demonstrated that *BDNF* reduction resulted in reduced neurodegeneration in animal DR models.^28^ Hypoxia-induced retinal neovascularization was shown to result in DR and vision loss. One study showed that oxygen-induced retinal vessel loss and neuronal degeneration occurred in *IGFBP3*-deficient mice.^29^ Our analysis put forth that *IGFBP3 *gene expression highly decreased (−6.50-fold change) in DR patients and it showed that false regulation of this gene could be related to the disease progression and diagnosis.

In recent years, the roles of SERPING1 and IGFBP3 have garnered increasing attention as potential therapeutic targets for various diseases, including autoimmune disorders and cancers. SERPING1, an important inhibitor of the classical complement pathway, has been implicated in the regulation of immune responses and the prevention of excessive inflammation. Dysregulation of SERPING1 has been associated with conditions such as hereditary angioedema and inflammatory diseases.^30^ On the other hand, IGFBP3 is involved in cell proliferation, apoptosis, and tissue remodeling, making it a key player in cancer progression, tissue repair, and metabolic diseases. Emerging evidence suggests that manipulating the expression or activity of these proteins could provide novel therapeutic strategies. For example, targeting SERPING1 to modulate complement activation could be beneficial in treating autoimmune conditions, while IGFBP3 could be explored as a therapeutic agent in cancer and metabolic disorders due to its regulatory influence on cell growth and survival.^31^ Future studies are needed to further elucidate the mechanistic pathways underlying these proteins’ roles and evaluate their potential in clinical applications.

Our study focused on gene expression analysis to explore new targets for disease therapy and diagnosis. In this study, we proposed target genes for the diagnosis and treatment of DR. The 2 main limitations of this investigation are the proportionally small number of enrolled patients and the inability to classify DR cases as non-proliferative DR and proliferative DR. Despite these limitations, our findings indicated that the *HIF1A* and *IGFBP3* genes could be used to confirm retinopathy disease progression in patients with DM. The present study showed that higher upregulation of the *HIF1A *gene and downregulation of the *IGFBP3 *gene could result in the transformation of DM into DR pathogenesis. At least, these genes could confirm DR occurrence in the early stages of disease progression. Moreover, these genes might be used to treat patients at the molecular level without utilizing other surgical or medical applications. Nevertheless, further *in vivo* animal experiments should be constructed with these genes to determine specific application strategies for DR.

## Figures and Tables

**Table 1.** Baseline Characteristics of Participants

Patients	HbA1C	Cholesterol	Triglyceride	HDL	LDL	Diabetes Duration
(−) Control	4.86 ± 0.43	146.14 ± 51.72	119.78 ± 45.49	36.92 ± 6.12	103.28 ± 33.55	7.54 ± 1.51
Diabetes	10.19 ± 1.31	199.25 ± 51.73	250.06 ± 48.22	39.18 ± 4.83	133.18 ± 35.58	8.06 ± 2.09
Diabetic retinopathy	10.28 ± 2.47	246.81 ± 58.61	316.54 ± 37.78	47.09 ± 12.23	177.27 ± 30.35	

HbA1C, hemoglobin A1C; HDL, high-density lipoprotein; LDL, low-density lipoprotein.

**Table 2.** Primer Pairs Used in Real-Time Polymerase Chain Reaction Analysis

Gene	Forward Primer	Reverse Primer	References
*BDNF*	5-CAGGGGCATAGACAAAAG-3	5-CTTCCCCTTTTAATGGTC-3	^32^
*CX3CR1*	5’-GGGCCTGAGCCAAGCTAGAA-3’	5’-ACAGCACCTTCCAGGGATGG-3’	^33^
*HIF1A*	5’-TTCACCTGAGCCTAATAGTCC-3’	5’-CAAGTCTAAATCTGTGTCCTG-3’	^34^
*IGFBP3*	5’- GGTGTCTGATCCCAAGTTCC- 3’	5’- CGGAGGAGAAGTTCTGGGTA- 3	^35^
*STAT3*	5’- ACCCAACAGCCGCCGTAG-3’	5’- CAGACTGGTTGTTTCCATTCAGAT-3’	^36^
*VEGFA*	5’- CTTGCCTTGCTGCTCTACC-3'	5’- CACACAGGATGGCTTGAAG- 3'	^37^
*TIMP3*	5’-TATGACTAGTAGCCCAGTGATGCTTGTGTTG-3’	5’- TATGAAGCTTATTCAGGAAAATGGCGGCATGTG-3’	^38^
*SERPING1*	5’-ATTCTCCTACCCAGCCCACT-3	5’-GGCGTCACTGTTGTTGCTTA-3’	^22^

**Table 3.** Power Analysis to Confirm Sample Size for the Gene Expression Investigation via G*Power Software (α = 0.05, 1−β = 0.8)

Genes	Number of Samples to Reach the Power of 0.8
*HIF1A*	4
*SERPING1*	4
*VEGFA*	4
*CX3CR1*	4
*BDNF*	10
*IGFBP3*	4
*TIMP3*	6
*STAT3*	8

## Data Availability

The data that support the findings of this study are available on request from the corresponding author.
